# Low Vitamin B12 Levels among Newly-Arrived Refugees from Bhutan, Iran and Afghanistan: A Multicentre Australian Study

**DOI:** 10.1371/journal.pone.0057998

**Published:** 2013-02-28

**Authors:** Jill Benson, Christine Phillips, Margaret Kay, Murray T. Webber, Alison J. Ratcliff, Ignacio Correa-Velez, Michelle F. Lorimer

**Affiliations:** 1 Discipline of General Practice, University of Adelaide, South Australia, Australia; 2 Social Foundations of Medicine, Medical School, Australian National University, Acton, Australia; 3 Discipline of General Practice, The University of Queensland, Brisbane, Queensland, Australia; 4 Hunter New England Health Refugee Health Program, HNE Local Health District, New South Wales, Australia; 5 Royal Hobart Hospital, Tasmania, Australia; 6 School of Public Health and Social Work, Queensland University of Technology, Brisbane, Queensland, Australia; 7 Data Management and Analysis, University of Adelaide, South Australia, Australia; Johns Hopkins Bloomberg School of Public Health, United States of America

## Abstract

**Background:**

Vitamin B12 deficiency is prevalent in many countries of origin of refugees. Using a threshold of 5% above which a prevalence of low Vitamin B12 is indicative of a population health problem, we hypothesised that Vitamin B12 deficiency exceeds this threshold among newly-arrived refugees resettling in Australia, and is higher among women due to their increased risk of food insecurity. This paper reports Vitamin B12 levels in a large cohort of newly arrived refugees in five Australian states and territories.

**Methods:**

In a cross-sectional descriptive study, we collected Vitamin B12, folate and haematological indices on all refugees (n = 916; response rate 94% of eligible population) who had been in Australia for less than one year, and attended one of the collaborating health services between July 2010 and July 2011.

**Results:**

16.5% of participants had Vitamin B12 deficiency (<150 pmol/L). One-third of participants from Iran and Bhutan, and one-quarter of participants from Afghanistan had Vitamin B12 deficiency. Contrary to our hypothesis, low Vitamin B12 levels were more prevalent in males than females. A higher prevalence of low Vitamin B12 was also reported in older age groups in some countries. The sensitivity of macrocytosis in detecting Vitamin B12 deficiency was only 4.6%.

**Conclusion:**

Vitamin B12 deficiency is an important population health issue in newly-arrived refugees from many countries. All newly-arrived refugees should be tested for Vitamin B12 deficiency. Ongoing research should investigate causes, treatment, and ways to mitigate food insecurity, and the contribution of such measures to enhancing the health of the refugee communities.

## Introduction

Among countries with formal refugee resettlement programs, Australia ranks third after the U.S. and Canada in the size of the annual intake of refugees, with 13750 refugees entering in 2010/11. The Australian government has increased the refugee resettlement program to 20,000 places in 2012/13. Refugees are a heterogeneous population who share the experience of being displaced through conflict and human rights abuse in their home countries. Chronic food insecurity – the inability to ensure basic nutritional needs of the population – has emerged as one of the pressing problems resulting from, and in turn reinforcing, political instability in many refugee-source countries [1] . Many refugees who arrive in Australia through the humanitarian program come from countries at extreme and moderate risk of food insecurity (Table 1).

**Table 1:** Top ten source countries for refugees in the Australian off-shore resettlement program and risk of food insecurity.

	2010		2011	
	Top ten refugee-source countries, Australian off-shore humanitarian intake [37]	Risk of food insecurity [38]	Top ten refugee-source countries, Australian off-shore humanitarian intake [39]	Risk of food insecurity [40]
1	Burma	Moderate	Iraq	Low
2	Iraq	Low	Burma	Moderate
3	Bhutan	Moderate	Afghanistan	Extreme
4	Afghanistan	Extreme	Bhutan	Moderate
5	Congo (DRC)	Extreme	Congo (DRC)	Extreme
6	Ethiopia	Extreme	Ethiopia	Extreme
7	Somalia	Extreme	Sri Lanka	Moderate
8	Sudan	Extreme	Iran	Low
9	Liberia	Extreme	Sudan	Extreme
10	Sierra Leone	Moderate	Somalia	Extreme

Nutritional deficiencies among newly arrived refugees, particularly iron and Vitamin A deficiency, have been well-described in the national and international literature [2], [3], [4]. Refugees from countries with compromised food supplies, and particularly those where intake of animal source food (ASF) is limited, are also at risk of Vitamin B12 deficiency. Although there is a deficit of national data, surveys in some refugee source countries, such as Iran [5], Nepal [6], [7] and Kenya [8] (where many South Sudanese refugees live) have demonstrated high rates of Vitamin B12 deficiency.

Despite this, Vitamin B12 is not part of most recommended screening protocols for newly arrived refugees [9]. Emerging data suggest that at least some refugee populations may be at particular risk of Vitamin B12 deficiency. In a study of 326 refugees undertaking post-arrival screening in Minnesota, Texas and Utah, 27% of those from Bhutan and 12% from Somalia had Vitamin B12 deficiency [10]. Case study data in Australia has demonstrated extremely low Vitamin B12 levels without associated macrocytosis in some newly arrived refugee patients [11]. The U.S. three-state study [10] is the largest study yet reported on Vitamin B12 deficiency in newly arrived refugees in a resettlement country, but one-third of this population were from Burma, and with the exception of Somalia, African countries were under-represented.

Although no internationally agreed threshold exists above which a prevalence of low Vitamin B12 is indicative of a population health problem, McLean and colleagues [12] used a threshold of 5% in their review of folate and Vitamin B12 deficiencies worldwide. Accordingly, we hypothesised that Vitamin B12 deficiency is greater than 5% among newly arrived refugees, and that prevalence might be higher among women, who may have reduced access to ASFs. There are precedents for systematic under-recognition of significant health issues among newly arrived refugees. For example, the high prevalence of Vitamin D deficiency among refugees was under-recognised, or often misdiagnosed as somatisation, for many years in resettlement countries [13], [14], resulting in long delays in the development of policy approaches to manage this health risk. Emerging health problems among refugees can be missed because they are resettled into small geographically diverse populations, demographic indicators that might reliably identify refugees on datasets are lacking, and overworked health care services often have little capacity to undertake service-based research [15], [16]. The Refugee Health Network of Australia (RHeaNA) was established to share information across all service providers who work in refugee health, allowing rapid identification and response to emerging health problems [16].

In this paper we report the prevalence of low Vitamin B12 levels across newly arrived refugees in five states and territories in Australia.

## Methods

### Ethics Approval

This study was approved by the Human Research Ethics Committees of: Australian National University, Hunter New England Health Local Health District, Mater Health Services, South Australian Department of Health, Tasmania Department of Health and Human Services, and University of Adelaide.

### Participating Sites

Australia uses a decentralised model for resettlement of refugees, and does not have a central national health screening service, as for example, New Zealand does. Initial screening of refugees is voluntary, and conducted by general practitioners working in community health services, hospital-based health assessment services, NGOs or private general practices. Participating services and groups in this study were the following members of the Refugee Health Network of Australia: the Migrant Health Service (Adelaide, South Australia), Companion House Medical Service (Canberra, ACT), Refugee Health Queensland (Brisbane), Refugee and Humanitarian Arrival Clinic (Royal Hobart Hospital, Tasmania), Migrant Resource Clinic (Launceston, Tasmania) and the Hunter New England Refugee Health Program (Newcastle, NSW).

### Study Population

Refugees from all age groups who arrived in Australia on humanitarian visas, with residence in Australia of less than 12 months, and who attended one of the participating refugee health centres between 1 July 2010 and 21 July 2011.

### Data Collection

All refugees who presented to one of these services were asked for their consent for screening. In the case of children, consent was sought from their parent or adult guardian. An information sheet outlining the importance of Vitamin B12 and the purpose of the data collection was interpreted verbally for each patient by a professional interpreter. Baseline demographic data included date of birth, gender, date of arrival in Australia, date of test, country of birth and cultural identity. As part of the usual testing arranged for newly-arrived refugees, these health services collected haemoglobin (Hb), mean corpuscular volume (MCV), Vitamin B12 levels and folate levels. Results were collated and de-identified by the clinicians at each site, then provided to the central researcher (JB).

### Definitions

The World Health Organisation (WHO) has recommended that a level of <150 pmol/L (<203 pg/mL) be used as the threshold for defining Vitamin B12 deficiency [17]. This measure was chosen for its clinical relevance, since levels below 150 pmol/L are associated with health consequences. Other measures that might confirm true Vitamin B12 deficiency such as holotranscobalamin, homocysteine or methylmalonic acid (MMA) are expensive and are not used in the clinic setting [17]. Anaemia was defined as a haemoglobin <120 g/L.

### Measurement

Serum Vitamin B12 levels were measured using the Abbott Architect automated assay in five sites, and the Beckman Unicel DxI 800 Analyser in one site. Full blood count, including MCV, was measured using automated machines at each site which have been evaluated and approved by the National Association of Testing Authorities (NATA).

### Analysis

For analysis Vitamin B12 category was dichotomised (<150 pmol/L, ≥150 pmol/L). Where appropriate, chi square analysis was used for binary categorical variables. For the high prevalence countries, the probability of Vitamin B12 deficiency was modelled using separate logistic regressions for age and gender. All analyses were tested for significance at the 5% level. Odds ratios (and 95% confidence intervals) are presented for each predictor of interest.

## Results

**Figure 1:**
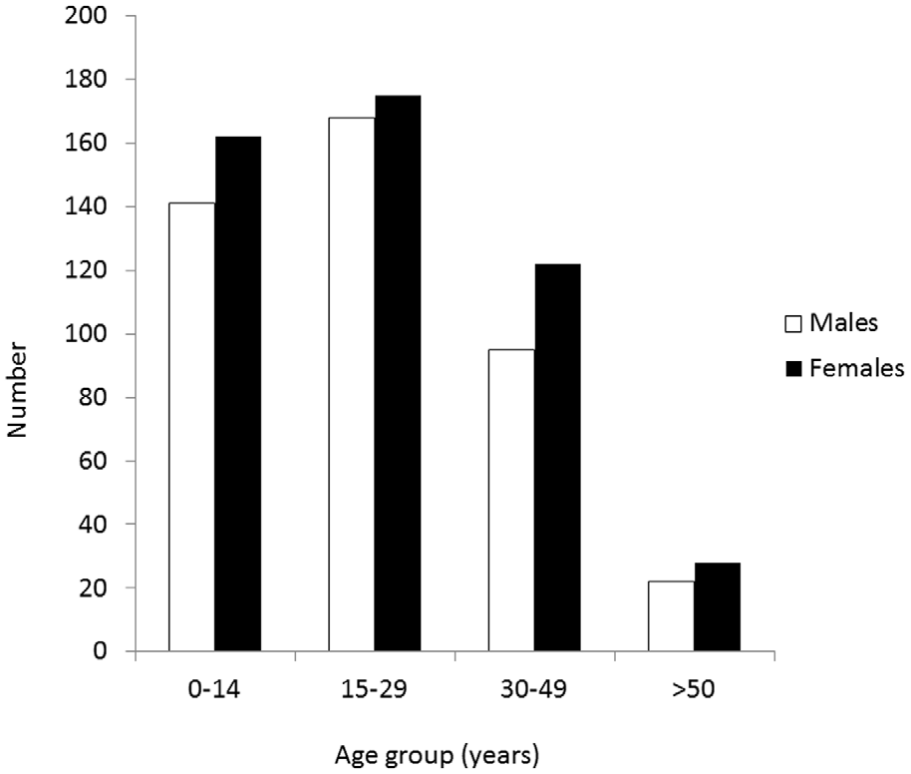
Age and gender distribution of newly arrived refugees who participated in the study (n = 916).

The study population consisted of 916 persons (427 females, 489 males). Two were excluded as there was no date of birth. Fifty-four persons declined to be screened, or did not have Vitamin B12 tested, resulting in a participation rate of 94%. The age distribution of the population by gender is presented in Figure 1. Compared to the overall population of humanitarian arrivals to Australia during the study period, there is a slight over-representation of people under 30 years of age in our sample (71%, compared to 63% in the national sample) and women (47%, compared to 42% in the national sample) [18]. Most participants were examined shortly after they arrived in Australia. Sixty-six per cent had arrived in Australia within the previous month, with only 6% having a duration of stay recorded of more than four months.

### Vitamin B12 Deficiency

Overall16.5% of newly arrived refugees had results consistent with Vitamin B12 deficiency (<150 pmol/L). Low levels of Vitamin B12 were least prevalent among those under 14 years of age (11.5%), compared to those aged 15–29 years (18.3%), 30–49 years (19.9%) and over 50 years (19.6%) (p<0.001;). Fourteen per cent of females and 18.6% of males in the cohort had levels below 150 pmol/L (p = 0.006). Among women of reproductive age (>15 years) 17% of had Vitamin B12 deficiency, though contrary to expectation older males (19.6%) also had Vitamin B12 deficiency.

Persons from Bhutan, Iran and Afghanistan had the highest rates of low Vitamin B12, with approximately one-third of participants from Bhutan and Iran and one-quarter from Afghanistan having levels below 150 pmol/L (Table 2). There was a significant association between Vitamin B12 deficiency and advancing age in Bhutan, but not in Afghanistan or Iran (Table 3). In addition, there was no association between Vitamin B12 and gender for any of these high prevalence countries (Table 3).

**Table 2:** Country of origin of refugees and Vitamin B12 results.

Country of origin	Total number of refugees	Vitamin B12 level			Median Vitamin B12 level	Interquartile range for B12 levels
		<150 pmol/L	150 – 240 pmol/L	>240 pmol/L		
		n (%)	n (%)	n (%)		
Afghanistan	159	39 (24.5)	61(38.4)	59 (37.1)	207	150, 291
Bhutan	196	61 (31.1)	82 (41.8)	53 (27.0)	188	137.5, 249
Burma	113	2 (1.8)	8 (7.1)	103 (91.2)	412	312, 532
Iraq	70	14 (20)	27 (38.6)	29 (41.4)	216	164, 308
Iran	48	14 (29.2)	17 (35.4)	17 (35.4)	176	145.5, 255.5
Sri Lanka	23	0	5 (21.7)	18 (78.3)	315	242, 373
Horn of Africa^1^	104	11 (10.6)	37 (35.6)	56 (53.9)	250.5	187, 338
Central Africa^2^	145	5 (3.5)	23 (15.9)	117 (80.7)	374	282, 510
West Africa 3	13	0	1 (7.7)	12 (92.3)	492	399, 701
East Africa^4^	32	3( 9.4)	6 (18.8)	23 (71.9)	355.5	236.5, 440
Other^5^	13	2 (15.4)	2 (15.4)	9 (69.3)	267	208, 288

1 Includes: Somalia, Ethiopia, Eritrea.

2 Includes: Congo (DRC), Rwanda, Burundi.

3 Includes: Sierra Leone, Liberia.

4 Includes: Sudan, Kenya.

5 Includes: Bangladesh, Pakistan, China (Uyghur), India, Zimbabwe.

**Table 3:** Low Vitamin B12 levels by age and gender, respectively, for each high prevalence country.

	Bhutan (n = 196)		Iran (n = 48)		Afghanistan (n = 159)	
	<150 pmol/L	Odds Ratio (95% CI)	<150 pmol/L	Odds Ratio (95% CI)	<150 pmol/L	Odds Ratio (95% CI)
Age Category	P = 0.048		P = 0.54		P = 0.32	
0–14 years	16.1%	1 (Ref)	23.1%	1 (Ref)	34.9%	1 (Ref)
15–29 years	35.8%	2.92 (1.22, 6.96)	25.0%	1.11 (0.22, 5.73)	21.8%	0.52 (0.23, 1.19)
30–49 years	38.5%	3.26 (1.32, 8.08)	40.0%	2.22 (0.43, 11.60)	18.8%	0.43 (0.15, 1.28)
≥ 50 years	38.1%	3.21 (1.04, 9.98)	0	0	16.7%	0.37 (0.04, 3.50)
Gender	P = 0.40		P = 0.62		P = 0.18	
Female	28.1%	1 (Ref)	33.3%	1 (Ref)	30.9%	1 (Ref)
Male	33.6%	1.30 (0.70, 2.39)	26.7%	0.73 (0.20, 2.59)	21.2%	0.60 (0.29, 1.26)

### Anaemia and Red Cell Indices

Results of full blood counts were available for 913/916 participants. Of these, 16.4% had anaemia, with eight having haemoglobin levels below 90 g/L. Patients with Vitamin B12 deficiency were no more likely to have anaemia than patients without deficiency. The prevalence of anaemia (Hb<120 g/L) in the study population was 18.5% (28/151) in cases with Vitamin B12<150 pmol/L, and 16.0% (122/762) in cases with levels ≥150 pmol/L (p = 0.5). Macrocytosis (MCV>95 fL) was reported in 4.6% (7/151) of cases who also had Vitamin B12<150 pmol/L, and 0.9% (7/762) of cases with Vitamin B12 levels ≥ 150 pmol/L (p = 0.004), indicating a sensitivity of 4.6% for macrocytosis in detecting Vitamin B12 deficiency. None of the cohort had folate deficiency.

## Discussion

This study has shown that Vitamin B12 deficiency is prevalent among newly-arrived refugees to Australia, particularly those from Bhutan, Iran and Afghanistan. Low Vitamin B12 levels were also more prevalent in males, and in older age-groups in some countries. Few population-based surveys have assessed Vitamin B12 status throughout the world and this dearth of data has made it difficult to estimate the magnitude of Vitamin B12 deficiency at both regional and global levels [12]. Nationally representative surveys have reported high prevalence of Vitamin B12 deficiency among children aged 1 to 6 years in Mexico (7.7%) [19], school-aged children in Venezuela (11.4%) [20], women of reproductive age in Germany (14.7%) [21], Vietnam (11.7%) [22], and the United Kingdom (11%) [23], pregnant women in Venezuela (10.9%) [20], and in the elderly in New Zealand (12%) [24]. There are no nationally representative surveys for Australia. The very few surveys that have investigated this issue in refugee source countries have been based on local- or district-level data. These surveys have reported high prevalence of Vitamin B12 deficiency among school-aged children in Kenya (where many Sudanese refugees live) (40%) [25], and pregnant women in Nepal (where Bhutanese refugees have lived since the early 1990 s), ranging from 28% [26] to 49% [6].

To our knowledge, only one study has previously investigated Vitamin B12 status in a refugee population [10]. It found Vitamin B12 deficiency in 64% (63 of 99) of specimens obtained during overseas medical examinations from adult Bhutanese refugees, 27% (17 of 64) of post-arrival medical screenings collected by three state health departments in the U.S., and 32% (19 of 60) of resettled Bhutanese refugees screened at a health clinic in the U.S. The study also reported post-arrival serum Vitamin B12 concentrations among 326 resettled refugees from 12 countries of origin, including Bhutan. Other than the Bhutanese, only refugees from Somalia were found to have Vitamin B12 deficiency (10 of 82, or 12%) [10]. Our study, which is based on a larger sample (n = 916) and is drawn from large community-based screening services for newly-arrived refugees distributed across five of the seven states and territories in Australia, confirms the U.S. findings of the high prevalence of Vitamin B12 deficiency in Bhutanese refugees, and provides further information on deficiency among patients from Iran and Afghanistan. This study also confirms that very few refugees with Vitamin B12 deficiency have macrocytosis and therefore macrocytosis is of little use as a tool for screening for Vitamin B12 deficiency in this population.

Despite food insecurity in their country of origin, our African participants reported lower prevalence of Vitamin B12 deficiency compared to participants from Bhutan, Iran and Afghanistan. High levels of anaemia and micronutrient deficiencies found in refugee camps, including Kakuma refugee camp in Kenya, has led the United Nations World Food Program (WFP) and UNHCR to improve the quality of the diet available including the addition of micronutrient powders (MNP) [27]. A MNP sachet (1 g) contains 0.9 µg of Vitamin B12. Our findings suggest that these programs are having a positive impact on reducing Vitamin B12 deficiency and should be a common practice in refugee camps worldwide.

The Bhutanese customarily do not consume animal source foods and supplementation may be necessary for this population. Although people from Iran and Afghanistan do consume meat, many of those who have fled their countries of origin due to war and human rights violations have not been in refugee camps and so would not have received micronutrient supplementation. It is possible that their Vitamin B12 levels may return to normal in a country with ready access to a wide variety of foods.

However, it should not be assumed that residence in Australia will necessarily result in a rapid improvement in dietary quality and quantity. In a study of 31 refugees who had settled in Perth, Gallegos and co-authors [28] found that 71% had experienced ‘running out of food’. A 2007 study also found that immigrant women who have spent less than half their life in the U.S. were at higher risk of food insecurity [29].

Our results raise the question of whether or not refugees with Vitamin B12 deficiency who do not report symptoms should be treated. Since their study, the Center for International Health in Minnesota has developed a policy of administering Vitamin B12 500–1000 mcg orally to all asymptomatic Bhutanese patients. All other newly arrived refugees have their Vitamin B12 tested and where levels are low or borderline, a protocol for oral treatment is followed (personal communication Dr Ann Settgast and Dr Michael Westerhaus, Center for International Health, Minnesota).

Low maternal levels of Vitamin B12 appear to be an independent risk for neural tube defects [30]. A recent finding in South Australia is an increase in the prevalence of neural tube defect affected pregnancies in women from the Middle East and South and Central Asia (personal communication Dr Wendy Scheil, Public Health Physician, Head Pregnancy Outcome Unit, SA Health, Government of South Australia). There is an evolving debate about whether Vitamin B12 should be added to folic acid as a supplementation, or be routinely checked for and supplemented in pregnancy [31], [32], [33].

Recently it has been argued the measurement of Vitamin B12 levels lacks sensitivity or specificity and that biomarkers such as elevated levels of methylmalonic acid (MMA) and homocysteine, two Vitamin B12-dependent enzymes, are a more sensitive measure of Vitamin B12 deficiency [34], although this is still subject to much debate [35]. It is possible that the true rate of subclinical Vitamin B12 deficiency, if measured using biomarkers, may be significantly greater than reported here.

Our study has a number of limitations. First, this is a cross-sectional descriptive study that has assessed Vitamin B12 status on newly-arrived refugees from all age groups who attended one of the five collaborating refugee health services over a one year period. Although we cannot claim that our sample is representative of all humanitarian arrivals to Australia during the study period, the study involved participants from five of the seven Australian states and territories, and had a high participation rate of 94% of eligible participants. Importantly, the population described in our study is broadly representative of the major countries of origin of refugees resettled across the UNHCR’s resettlement program. In 2011, UNHCR reported that the leading countries of origin for refugees seeking resettlement were Iraq, Burma, Bhutan, Somalia, the Democratic Republic of Congo, Iran and Afghanistan [36]. Our findings are therefore of relevance to other resettlement countries. Second, we have not reported clinical examination findings of patients with Vitamin B12 deficiency, as standardising examination protocols across the refugee health services was beyond the scope of the study. The recent U.S. three-state study was triggered by reports that physicians were seeing an increased number of patients with peripheral neuropathy. Of the 141 Bhutanese refugees seen at the refugee clinic in St Paul Minnesota, 60 were tested for Vitamin B12 levels, 19 (32%) were Vitamin B12 deficient, and two (11%) had peripheral neuropathy [10]. Third, in dichotomising B12 levels into low (<150 pmol/L) and normal (≥150 pmol/), we may have simplified a complex picture; Vitamin B12 levels between 150–240 pmol/L are borderline results which some authors have argued may also include patients with true Vitamin B12 deficiency [34].

Policies to enhance the health of refugees after resettlement, including screening, require evidence. There are structural difficulties in gathering data that can inform clinical practices for small, vulnerable populations who are not readily identifiable in standard datasets. A further challenge for refugee health policymakers is that the refugee populations resettled in Australia constantly change in response to international geopolitical events. Individual refugee services are usually too small and overworked to produce datasets of sufficient size and quality to demonstrate emerging conditions among refugee populations. Both the U.S. and our Australian studies investigating Vitamin B12 deficiency among resettled refugees were initially triggered by clinicians’ observations in a few services, but confirmed by the combined data of a network of services working together for a common purpose. Refugees, like other vulnerable populations, require networks of services that can undertake rapid research on emerging conditions, and guide policymakers on the appropriate responses to meet these emerging conditions.

Although a health assessment is recommended as soon as possible after a refugee resettles in their host country, evidence for best practice in the delivery of refugee health care to newly-arrived refugees is still in its infancy. Currently there is no consensus about Vitamin B12 testing in this population though this has been performed in a few centres in Australia. This multicentre study has shown that low Vitamin B12 levels are common in newly arrived refugees from a number of countries. Ongoing research is needed to investigate effective approaches to treat Vitamin B12 deficiency and mitigate food insecurity, and to understand the contribution of such measures to enhancing the health of the refugee communities.
